# Comparison of patient comprehension of rapid HIV pre-test fundamentals by information delivery format in an emergency department setting

**DOI:** 10.1186/1471-2458-7-238

**Published:** 2007-09-12

**Authors:** Roland C Merchant, Erin M Gee, Melissa A Clark, Kenneth H Mayer, George R Seage, Victor G DeGruttola

**Affiliations:** 1 Department of Emergency Medicine, Warren Alpert Medical School of Brown University, Providence, Rhode Island, USA; 2 Department of Community Health, Warren Alpert Medical School of Brown University, Providence, Rhode Island, USA; 3 Department of Epidemiology, Harvard School of Public Health, Boston, Massachusetts, USA; 4 Department of Medicine, Division of Infectious Diseases, Warren Alpert Medical School of Brown University, Providence, Rhode Island, USA; 5 Department of Biostatistics, Harvard School of Public Health, Boston, Massachusetts, USA

## Abstract

**Background:**

Two trials were conducted to compare emergency department patient comprehension of rapid HIV pre-test information using different methods to deliver this information.

**Methods:**

Patients were enrolled for these two trials at a US emergency department between February 2005 and January 2006. In Trial One, patients were randomized to a no pre-test information or an in-person discussion arm. In Trial Two, a separate group of patients were randomized to an in-person discussion arm or a Tablet PC-based video arm. The video, "Do you know about rapid HIV testing?", and the in-person discussion contained identical Centers for Disease Control and Prevention-suggested pre-test information components as well as information on rapid HIV testing with OraQuick^®^. Participants were compared by information arm on their comprehension of the pre-test information by their score on a 26-item questionnaire using the Wilcoxon rank-sum test.

**Results:**

In Trial One, 38 patients completed the no-information arm and 31 completed the in-person discussion arm. Of these 69 patients, 63.8% had twelve years or fewer of formal education and 66.7% had previously been tested for HIV. The mean score on the questionnaire for the in-person discussion arm was higher than for the no information arm (18.7 vs. 13.3, p ≤ 0.0001). In Trial Two, 59 patients completed the in-person discussion and 55 completed the video arms. Of these 114 patients, 50.9% had twelve years or fewer of formal education and 68.4% had previously been tested for HIV. The mean score on the questionnaire for the video arm was similar to the in-person discussion arm (20.0 vs. 19.2; p ≤ 0.33).

**Conclusion:**

The video "Do you know about rapid HIV testing?" appears to be an acceptable substitute for an in-person pre-test discussion on rapid HIV testing with OraQuick^®^. In terms of adequately informing ED patients about rapid HIV testing, either form of pre-test information is preferable than for patients to receive no pre-test information.

## Background

According to CDC estimates, there were 1,039,000–1,185,000 persons living with an HIV infection in the United States as of 2003 [1]. Of these, at least 25% are infected with HIV and are unaware of their infection. To help those infected with HIV learn their HIV status, the Centers for Disease Control and Prevention (CDC) advocate expanding HIV testing in non-traditional test settings, such as emergency departments (EDs) [2,3]. Rapid HIV testing might further reduce the number of persons unaware of their HIV status by streamlining the HIV testing process and by making HIV testing more readily available in a variety of locations. In September 2006, the CDC released new guidelines on HIV testing for healthcare settings [3]. To help facilitate HIV testing, the CDC recommended that traditional pre-test counseling be simplified by separating the routine requirement for pre-test information from the as needed provision of prevention counseling.

One limitation of HIV testing in non-traditional settings is the difficulty of effectively providing pre-test information to those undergoing testing. In a busy ED, it can be challenging for staff to adequately give patients CDC-recommended components of HIV pre-test information. The open-space environment of EDs, the numerous distractions and noises, the sometimes critical nature of patient visits, and staff time limitations make it difficult to educate patients about HIV and HIV testing. In addition, staff knowledge about HIV testing varies, so information presented to patients might not be uniform and sometimes might not be accurate. Although written information is a potential alternative to an in-person discussion, this might not be adequate for all patients, given wide variations in patient reading abilities.

One approach to address limitations of staff availability and to ensure standardization of HIV pre-test information is through the use of a patient educational video in lieu of an in-person discussion. Videos have been recommended or used as an adjunct to pre-test information or as substitutes for in-person discussion for standard (as opposed to rapid) HIV testing [4-8]. However, there have been few studies evaluating their effectiveness in providing this information, particularly as compared to an in-person discussion. Calderon, et al. created a video for standard HIV testing at the Jacobi ED in New York in response to a lack of HIV counselors after normal business hours [9]. Using a questionnaire developed by the authors, they observed that patients watching the video demonstrated greater knowledge (a 7% higher mean score) about standard HIV testing than those receiving information from an HIV test counselor. To our knowledge, there have been no studies in any setting evaluating the utility of a rapid HIV educational video as a substitute for in-person discussions about rapid HIV testing.

Prior to the release of the new CDC HIV testing guidelines, we conducted two randomized controlled trials comparing different methods of delivering rapid HIV pre-test information to adult ED patients. We assessed patient comprehension of rapid HIV pre-test information using a questionnaire. In Trial One, we compared patient comprehension using this questionnaire among patients randomized to receive no pre-test information to those randomized to receive an in-person discussion about rapid HIV testing from an HIV test counselor. In Trial Two, we compared a separate group of patients who were randomized to an in-person discussion to those randomized to receive HIV pre-test information from the educational video, "Do you know about rapid HIV testing?"[10] The video and the in-person discussion contained the same CDC-recommended elements on pre-test HIV information as well as the same information specific to rapid HIV testing with OraQuick^®^. The goal of this pilot study was to evaluate these methods of pre-test information delivery prior to the conduct of a larger randomized controlled trial that included performing rapid HIV testing.

## Methods

### Study design

Two randomized controlled trials were conducted that examined different methods of rapid HIV testing information delivery. The trials were conducted sequentially. For both trials, participant understanding of rapid HIV testing fundamentals was evaluated using a questionnaire. The first trial compared participants who received no information to those who underwent an in-person discussion. The second trial compared participants who underwent an in-person discussion to those who watched an educational video. The Rhode Island Hospital institutional review board (IRB) approved the study. This study was a pilot to a larger trial comparing the video to the in-person discussion information delivery methods among patients undergoing rapid HIV testing with OraQuick^®^.

### Study population and eligibility

The two trials were performed at the Rhode Island Hospital ED, which is a large, urban, academic ED in the northeastern US. Patients eligible for the trials were ED patients 18-55-years-old who were not pregnant; not incarcerated or in home confinement; not known to be HIV infected; able to speak, read, and write in English; were not deaf; and were not critically ill, intoxicated, or presenting to the ED for a psychiatric problem. Participants could only be members of one trial.

### Study conduct

Trial One was conducted February-April 2005 and Trial Two between April 2005-January 2006. A research assistant (RA) conducted the trials weekdays during arbitrarily selected eight-hour shifts. The shifts began between 7 a.m. and 3 p.m. and ended between 3 p.m. and 11 p.m. For each trial, a research assistant (RA) randomly screened for study eligibility 50% of the medical records of patients who were present in the ED during the RA's shift. Before each shift, the RA used a random number generator to choose either the number 1 or 2 [11]. These numbers represented "odd" and "even" terminal medical record digits, respectively. The RAs screened only the "odd" or the "even" medical records for that shift as determined by this random selection scheme. The random selection was conducted to reduce the possibility that the RA might selectively choose patients to include in the study that could affect the outcome of the study, i.e., reduce selection bias.

The RAs screened the ED medical records in the ambulatory care and urgent care areas of the ED looking for patients who potentially met the study eligibility criteria. Patients in the critical care, substance abuse, and psychiatric evaluation areas of the ED were not screened for study inclusion. The demographic profile (age, gender, race/ethnicity, marital status, and insurance type) for every patient screened was recorded into the study database. The RAs also recorded the reasons for exclusion from the study and did not approach those who were ineligible by ED medical record review.

Patients who appeared to meet eligibility criteria by medical record review were approached by the RA. The RA briefly outlined the study purpose to the patient and asked for their verbal consent to confirm their demographic profile, study eligibility, and to ask additional questions about their HIV testing history and HIV status. Patients who declined to answer these questions or who were found to meet exclusion criteria during this brief interview (e.g., told the RA that they were HIV-infected) were excluded from the study. The RA recorded the reasons for their exclusion. Patients who met study eligibility were apprised of the study and invited to participate. Verbal agreement to participate was obtained.

In Trial One, patients were randomized to an in-person discussion on rapid HIV testing information arm or a no-information arm. The no-information arm received no rapid HIV pre-test information of any type. In Trial Two, patients were randomized to an in-person discussion arm or a video arm. The RA randomized patients for each trial using their penultimate medical record number. Odd-numbered patients were assigned to the in-person discussion while even-numbered patients were assigned the other arm, respective to the trial. Following the delivery of rapid HIV test information (or no information), the RA orally administered an identical questionnaire to all participants to assess their comprehension on the material presented. At the completion of the questionnaire, respondents were given an opportunity to discuss any questions or concerns about the material or topics presented or the study itself. At the completion of the study, participants were provided a copy of the written consent summary and a brochure on HIV and HIV testing that also gave a list of locations in the local area where they could be tested for HIV.

### Rapid HIV pre-test information delivery

**Figure 1:**
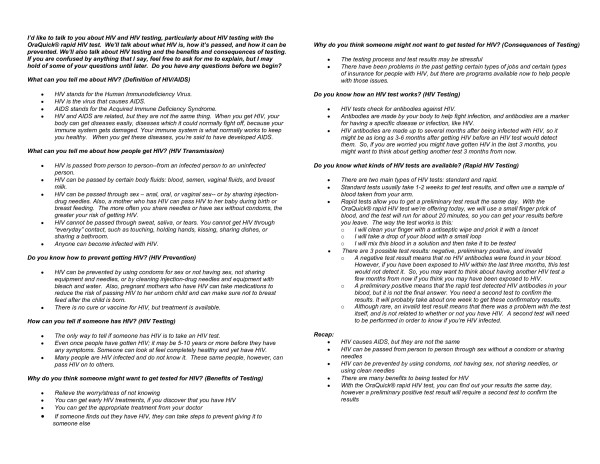
In-person discussion rapid HIV pre-test information outline.

For both trials, the in-person discussion was a semi-scripted conversation between the participant and RA on five topics: HIV and AIDS, HIV transmission, HIV prevention, HIV testing, and rapid HIV testing. An outline of the points covered in the in-person discussion is provided in Figure 1. The outline was created by the study authors and is based upon CDC recommendations for pre-test information [8] as well as manufacturer details on rapid HIV testing with OraQuick^® ^[12]. RAs conducting the study memorized the outline and were trained to deliver it through mock-interviews. The principal investigator observed the RAs during the trials, critiqued their performance, and corrected deviations from the outline. The RAs also underwent training by the state department of health to be certified as HIV test counselors.

The in-person discussion resembled a didactic presentation yet was conducted in a conversational style. Participants were asked an opening question for each of the five topics. The RA would endorse or correct the participant's response then state the points relevant to that topic---whether or not the participant answered the opening question correctly. To ensure uniformity of the presented information, the RA limited the discussion to the outline. The RAs had an abbreviated version of the outline in Figure 1 to use as a reference during the in-person discussion, but were not permitted read the outline verbatim to patients.

In Trial Two, the video information arm patients watched the 9.5 minute animated and live-action educational video "Do you know about rapid HIV testing?"[10] that contained the same rapid HIV pre-test information elements presented to the in-person discussion arm. The video and its development are described in the accompanying manuscript: Development of the rapid HIV testing video, "Do you know about rapid HIV testing?" [see Additional file 1]. Participants randomized to the video arm watched the video on a hand-held Tablet PC while awaiting medical care in the ED. They listened to the video using headphones provided by the RA.

### Assessment of participant comprehension of rapid HIV testing fundamentals

Participants in both trials were asked to complete the "HIV pre-test information comprehension" questionnaire to assess their comprehension of rapid HIV testing fundamentals. The development of the 26-item, "true", "false", "I don't know" questionnaire is described in the accompanying manuscript: Development of the "HIV pre-test information comprehension" questionnaire [see Additional file 2]. Participants in both trials received the identical version of the questionnaire. The RAs administered the questionnaire verbally to participants and recorded the responses onto a Tablet PC database. RAs provided all participants with a standardized, brief introduction to the nature of the questions, the reason for the questionnaire ("to determine how well we were able to give you this information about HIV and rapid HIV testing"), and the form of the responses needed ("true", "false", or "I don't know"). The RAs also informed participants that they would not be able to assist in answering the questions during the administration of the questionnaire, but could repeat questions as needed. Participants were asked to choose the response they thought was best, but to feel free to answer any question as "I don't know" when unsure of an answer. The RAs discussed the questions and answers with the participants after they completed the questionnaire.

### Data collection and analysis

The RAs recorded all study data (demographic and HIV test history, eligibility determinations, responses to the questionnaire, etc.) onto a Tablet PC study database using the QDS^® ^(NOVA Research, Bethesda, MD) data entry and management software. To ensure accuracy of the data entry, the data were entered in duplicate with immediate data verification. The data were analyzed using Stata 9.2 (Stata Corporation, College Station, TX). The analysis included tabulating the results of the medical record screening and eligibility determinations; participant demographic profiles, educational level, and HIV testing histories; and responses to the "HIV pre-test information comprehension" questionnaire. Patients were compared by their demographic profiles according to their eligibility, participation, and study arm assignment using Pearson's χ^2 ^test for categorical values; Fisher's exact test for categorical values with small samples; two-sample tests of binomial proportions for dichotomous values; and Wilcoxon rank-sum test for continuous values. Participant educational level and HIV testing history were compared by study arm assignment using Pearson's χ^2 ^test and Fisher's exact test.

The mean score and standard deviation, median, and range for each of the five topics areas of the questionnaire and the entire questionnaire were calculated by arm assignment for both trials. We originally estimated requiring a sample size of 55 for each arm of Trial Two to demonstrate a 12% absolute increase in mean scores. We assumed the same standard deviation for each arm, a normal distribution of scores, α = 0.05, β = 0.10, and use of Student's t-test. However, using plots of the scores for each trial and the Wilk-Shapiro test, we observed that the scores were not normally distributed, therefore, the scores for the arms were instead compared using the Wilcoxon rank-sum test. Differences were considered significant at the α = 0.05 level. In an exploratory analysis, the proportions of participants answering each question correctly were compared by arm assignment using two-sample tests of binomial proportions. This exploratory analysis was used to help identify sub-topics that might not have been well addressed in the pre-test information stage or highlight questions that might not have been well understood by participants.

## Results

### Study participant demography and HIV testing history

#### Trial One

**Figure 2:**
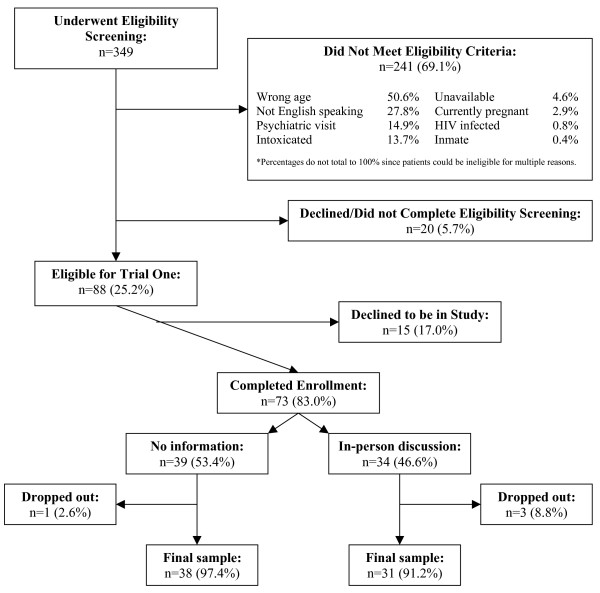
Trial one screening and enrollment diagram.

Figure 2 depicts the results of the screening and enrollment for Trial One. Of the 349 ED patients whose medical records were randomly screened for Trial One, 88 were eligible for participation. The primary reason for ineligibility was age < 18 or age > 55 years (50.6%). Twenty patients declined or did not complete the screening process. Of the 88 patients who completed the screening process with the RA and were eligible for the study, 73 originally agreed to be in the study. Of these, 34 patients were randomized to the in-person discussion and 39 to the no-information arm. Three people randomized to the in-person discussion arm and one in the no-information arm dropped out of the study.

Table 1 provides a comparison of the demography and HIV testing history of the patients by whether or not they participated in the study and by study arm. The participant and non-participant groups and the in-person discussion and no-information arms had similar demographic and HIV testing history profiles. Most of the participants were male, white, single/never married, had governmental insurance (Medicaid, Medicare, or both), had twelve or fewer years of formal education, and had previously been tested for HIV. Of those who had ever been tested for HIV, 40.4% had been tested within the prior year. The participants in the two arms were similar in their profiles except for partner status and time elapsed since their last HIV test. There were relatively more single/never married and separated participants in the no-information arm and relatively more married and divorced participants in the in-person discussion arm. There were relatively more participants who had been tested within the past six months in the no-information arm.

**Table 1:** Demographic and HIV testing history profiles: Trial One

	**Non-participants**	**Participants**	**p-value**	**In-person discussion arm**	**No information arm**	**p-value**
	*n = 15*	*n = 73*	*p*≤	n = 34*	n = 39*	*p*≤
**Median age (Range)**	40 (19–54)	35 (19–55)	0.27	38 (19–55)	34 (19–55)	0.14
	*%*	*%*		*%*	*%*	
**Gender**			0.45			0.60
Female	33.3	43.8		52.9	59.0	
Male	66.7	56.2		47.1	41.0	
**Ethnicity/Race**			0.75			0.96
Black	20.0	30.1		32.4	28.2	
Hispanic	6.7	5.5		5.9	5.1	
White	73.3	61.6		58.8	64.1	
Other	0.0	2.8		2.9	2.6	
**Partner status**			0.19			0.04
Single/never married	26.7	52.1		41.2	61.5	
Married	46.7	27.4		38.2	17.9	
Divorced	26.6	12.3		17.7	7.7	
Separated	0.0	5.5		0.0	10.3	
Unmarried couple	0.0	2.7		2.9	2.6	
**Insurance status**			0.46			0.09
Private	20.0	34.2		35.3	33.3	
Governmental	46.7	43.8		53.0	35.9	
Private/Governmental	6.6	1.4		2.9	0.0	
None	26.7	19.2		8.8	28.2	
Don't know	0.0	1.4		0.0	2.6	
**Years of formal education**			0.16			0.43
Grades 1–8	6.6	1.4		0.0	2.6	
Grades 9–11	6.7	21.9		17.7	25.6	
Grade 12 or equivalent	60.0	41.1		38.2	43.6	
College 1–3 years	26.7	21.9		23.5	20.5	
College 4 years	0.0	13.7		20.6	7.7	
**Prior HIV test**			0.19			0.99
Yes	53.3	64.4		64.7	64.1	
No	40.0	35.6		35.3	35.9	
Don't know	6.7	0.0		0.0	0.0	
**Time elapsed since last HIV test**	*n = 8*	*n = 47*	0.21	*n = 22*	*n = 25*	0.03
> 5 years	0.0	17.0		18.2	16.0	
> 2 years ≤ 5 years	37.5	21.3		31.8	12.0	
> 1 year ≤ 2 years	25.0	21.3		27.3	16.0	
> 6 months ≤ 1 year	0.0	14.9		18.2	12.0	
≤6 months	25.0	25.5		4.6	44.0	
Don't recall	12.5	0.0		0.0	0.0	

*Three patients in the in-person discussion and one in the no information arm dropped out from the study after randomization

#### Trial Two

**Figure 3:**
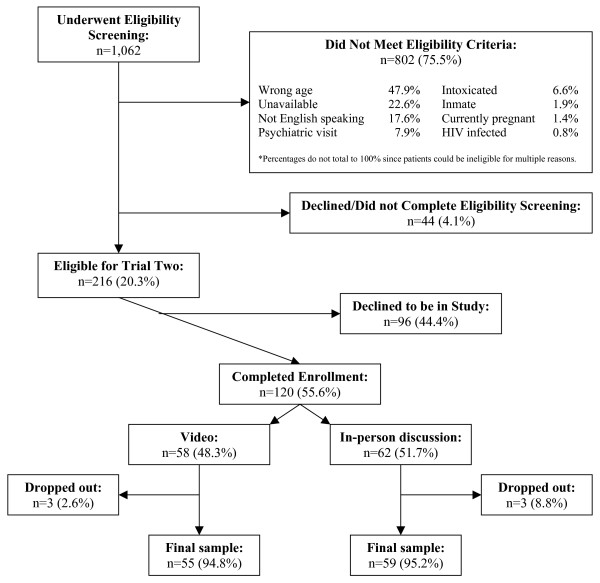
Trial two screening and enrollment diagram.

Figure 3 depicts the results of the screening and enrollment for Trial Two. Of the 1,062 ED patients whose medical records were randomly screened for Trial Two, 216 were eligible for participation. The primary reason for ineligibility was age < 18 or age > 55 years (47.9%). Forty-four patients declined or did not complete the screening process. Of the 216 patients who completed the screening process and were eligible for the study, 120 originally agreed to be in the study. Of these, 62 were randomized to the in-person discussion and 58 to the video arm. Three people randomized to the in-person arm and three to the video arm dropped out of the study.

As shown in Table 2, Trial Two participants and non-participants had similar demographic profiles. There were more non-participants who had not been previously tested for HIV. Most of the participants were male, white, single/never married, had private healthcare insurance, had twelve or fewer years of formal education, and had previously been tested for HIV. Of those tested for HIV, 43.2% had been tested within the past year. The two randomized arms were similar in their demographic and HIV history profiles.

**Table 2:** Demographic and HIV testing history profiles: Trial Two

	**Non-participants**	**Participants**	**p-value**	**Video arm**	**No information arm**	**p-value**
	*n = 96*	*n = 120*	*p*≤	n = 58*	n = 62*	*p*≤
**Median age (Range)**	38 (18–55)	35 (18–55)	0.21	34 (19–55)	37 (19–55)	0.45
	*%*	*%*		*%*	*%*	
**Gender**			0.62			0.48
Female	50.0	46.7		50.0	43.6	
Male	50.0	53.3		50.0	56.4	
**Ethnicity/Race**			0.69			0.09
Black	19.4	14.2		8.6	19.4	
Hispanic	11.2	11.7		8.6	14.5	
White	66.3	69.1		74.2	64.5	
Other	3.1	5.0		8.6	1.6	
**Partner status**			0.35			0.50
Single/never married	41.9	33.3		32.8	33.9	
Married	31.6	30.0		31.0	29.0	
Divorced	10.2	17.5		22.4	12.9	
Separated	5.1	4.2		3.5	4.8	
Widowed	1.0	0.0		0.0	0.0	
Unmarried couple	10.2	15.0		10.3	19.4	
**Insurance status**			0.99			0.48
Private	44.9	44.2		43.1	45.2	
Governmental	34.7	35.8		34.5	37.1	
Private/Governmental	1.0	1.7		0.0	3.2	
None	19.4	18.3		22.4	14.5	
**Years of formal education**			0.17			0.63
Grades 1–8	5.1	2.5		3.5	1.6	
Grades 9–11	30.6	18.3		17.2	19.4	
Grade 12 or equivalent	27.6	30.8		36.2	25.8	
College 1–3 years	24.5	34.2		32.8	35.5	
College 4 years	12.2	14.2		10.3	17.7	
**Prior HIV test**			0.02			0.23
Yes	55.1	68.3		65.5	71.0	
No	44.9	29.2		29.3	29.0	
Don't know	0.0	2.5		5.2	0.0	
**Time elapsed since last HIV test**	*n = 54*	*n = 81*	0.95	*n = 22*	*n = 25*	0.34
> 5 years	24.1	23.5		24.3	22.7	
> 2 years ≤ 5 years	22.2	17.3		16.2	18.2	
> 1 year ≤ 2 years	13.0	16.0		21.6	11.4	
> 6 months ≤ 1 year	16.7	19.7		10.8	27.3	
≤6 months	24.0	23.5		27.1	20.4	

*Three patients in each arm dropped out of the study after randomization

### Rapid HIV testing comprehension

Table 3 shows by arm assignment the percentages of participants correctly answering the "HIV pre-test information comprehension" questions. The results of the two-sample tests of binomial proportions are provided in an exploratory analysis to identify potential sub-topics or questions that might not have been well understood by participants. Across both trials, there were twelve questions correctly answered by more than 50% of the patients in all arms and five correctly answered by more than 75% of patients in all arms. Fewer than 50% of participants in all arms were able to correctly answer the questions regarding the role of HIV antibodies (question 15) and phlebotomy for the rapid HIV test (question 25).

**Table 3:** Correct responses on the "HIV pre-test information comprehension" questionnaire

		**Trial One**			**Trial Two**		
		**No information**	**In-person discussion**	**p-value**	**In person discussion**	**Video**	**p-value**
		*n = 31*	*n = 38*		*n = 59*	*n = 55*	
**Question**	**HIV/AIDS Definition**	*%*	*%*	p≤	*%*	*%*	p≤

1	If you were HIV infected, current drug treatments would let you live longer. (T)	86.8	90.3	0.65	83.1	89.1	0.36
2	People can get AIDS without getting HIV. (F)	36.8	71.0	0.00	86.4	76.4	0.17
3	Being infected with HIV does not mean you have AIDS. (T)	65.8	90.3	0.01	89.8	74.6	0.03
4	A person can be infected with HIV for 5 years or more without getting AIDS. (T)	76.3	96.8	0.01	91.5	92.7	0.81
	**HIV Transmission**						
5	A person cannot get HIV by donating blood. (T)	39.5	54.8	0.21	57.6	65.5	0.39
6	A woman with HIV can give HIV to her baby during breastfeeding. (T)	68.4	100.0	0.00	98.3	94.6	0.28
7	If someone gets HIV through needle sharing, that person can only spread HIV by sharing needles with other people. (F)	63.2	80.7	0.10	79.7	89.1	0.17
8	Coins, such as quarters or nickels, can carry HIV. (F)	68.4	93.6	0.01	98.3	90.9	0.08
9	A person cannot get HIV by putting their tongue in the mouth of someone who has HIV. (T)	42.1	61.3	0.11	67.8	81.8	0.08
	**HIV Prevention**						
10	HIV is destroyed by bleach. (T)	13.2	58.1	0.00	55.9	52.7	0.73
11	If you use injection drugs, the only way to prevent getting HIV is to quit using them. (F)	50.0	64.5	0.22	61.0	65.5	0.62
12	Wearing insect repellant to keep away mosquitoes will help prevent you from getting HIV. (F)	81.6	61.3	0.06	86.4	89.1	0.66
13	Not having sex is the only way to reduce your risk of getting HIV. (F)	71.1	74.2	0.77	71.2	74.6	0.68
14	You can prevent getting HIV after sex by washing your genitals or private parts. (F)	84.2	83.8	0.96	93.1	96.4	0.43
	**HIV Testing**						
15	HIV makes antibodies which harm a person's body. (F)	10.5	19.4	0.31	30.5	23.6	0.41
16	Having blood drawn for an HIV test will make you anemic. (F)	81.6	93.6	0.12	91.5	89.1	0.66
17	The HIV antibody test will help strengthen your antibodies to keep you from getting infected with HIV. (F)	76.3	83.9	0.43	83.1	85.5	0.73
18	If you were infected with HIV one week ago, your HIV test will be negative. (T)	42.1	61.3	0.11	57.6	80.0	0.10
19	The HIV antibody test will not tell you if you have AIDS. (T)	42.1	42.2	0.99	55.9	69.1	0.15
20	If your HIV test is negative, it must be repeated within a week to confirm the results. (F)	31.6	61.3	0.01	54.2	70.9	0.07
	**Rapid HIV Testing**						
21	It takes one to two days to perform a rapid HIV test. (F)	18.4	77.4	0.00	81.4	74.6	0.38
22	An invalid rapid HIV test result means you've been infected with HIV for fewer than 3 months. (F)	34.2	61.3	0.03	72.9	78.2	0.51
23	If your rapid HIV test is positive, then you will need a test to confirm this. (T)	71.1	87.1	0.09	81.4	89.1	0.25
24	The rapid HIV test with OraQuick uses a sample of your urine. (F)	34.2	90.3	0.00	81.4	87.3	0.39
25	A needle can be used to take blood from your arm for the OraQuick rapid HIV test. (T)	42.1	45.2	0.80	37.3	38.9	0.86
26	Even if your rapid HIV test is positive, you may not have HIV. (T)	39.5	61.3	0.07	54.2	63.6	0.31

Compared to the no-information arm in Trial One, the percentage of participants in the in-person discussion arm with correct answers to the questionnaire was significantly greater statistically for ten of the questions, but was greater in absolute value for twenty-three of the questions. Compared to the in-person discussion arm, the percentage of patients in the no-information arm was greater in absolute value for two questions and was the same as the in-person discussion arm for one question. There were two questions for which the percentage of correct responses was ≤50% in the in-person discussion arm as opposed to fourteen in the no-information arm.

Compared to the in-person discussion arm in Trial Two, the percentage of participants in the video arm with correct answers to the questionnaire was not significantly greater statistically for any of the questions, but was greater in absolute value for eighteen of the questions. The percentage of participants in the in-person discussion arm was significantly greater statistically than for participants in the video arm for one question (question 3), but was greater in absolute value for seven other questions. For both arms of the trial, there were two questions (questions fifteen and twenty-five) for which the percentage of correct responses was ≤50%.

Table 4 depicts the mean, median, and range of scores by arm assignment and subject area for the "HIV pre-test information comprehension" questionnaire. As shown for Trial One, participants in the in-person discussion arm had greater scores overall and had greater scores for each of the five subject areas than those in the no-information arm. The standard deviations for those in the no-information arm were also larger, which indicated a wide variation in ability to answer the questions among those in this arm. The difference in scores was largest for the rapid HIV testing section of the questionnaire. For Trial Two, there was a trend of higher scores in the video than the in-person discussion arm for three of the subject areas. Although the overall mean score appeared to be 4% larger for the video than the in-person discussion arm, there was no difference in these scores at our specified level of statistical significance and sample size using the Wilcoxon rank-sum test.

**Table 4:** Mean and median scores on the "HIV pre-test information comprehension" questionnaire

	**Trial One**					**Trial Two**				
	**No information**		**In-person discussion**		**p-value**	**In-person discussion**		**Video**		**p-value**
	*n = 38*		*n = 31*			*n = 59*		*n = 55*		

**Subject Area**	*μ (σ)*	*Median (range)*	*μ (σ)*	*Median (range)*	*p*≤	*μ (σ)*	*Median (range)*	*μ (σ)*	*Median (range)*	*p*≤

HIV/AIDS Definition	2.66 (1.15)	3 (0–4)	3.48 (0.68)	4 (1–4)	0.00	3.51 (0.68)	4 (1–4)	3.33 (0.86)	4 (1–4)	0.35
HIV Transmission	2.82 (1.18)	3 (1–5)	3.90 (0.94)	4 (2–5)	0.00	4.02 (0.96)	4 (2–5)	4.22 (0.79)	4 (2–5)	0.34
HIV Prevention	2.84 (1.37)	3 (0–5)	3.52 (1.03)	4 (2–5)	0.04	3.73 (1.19)	4 (1–5)	3.73 (1.10)	4 (1–5)	0.85
HIV Testing	2.84 (1.42)	3 (0–6)	3.65 (1.20)	4 (1–5)	0.01	3.73 (1.27)	4 (0–6)	4.18 (1.06)	4 (2–6)	0.05
Rapid HIV Testing	2.03 (1.38)	2 (0–5)	4.26 (1.12)	4 (2–6)	0.00	4.29 (1.25)	4 (2–6)	4.45 (1.14)	5 (1–6)	0.39
**All Subject Areas**	13.34 (4.45)	13 (6–24)	18.71 (3.50)	20 (10–24)	0.00	19.2 (3.63)	20 (11–25)	20.0 (2.98)	21 (11–25)	0.33

## Discussion

The CDC recently recommended routine, universal HIV screening in US primary care settings and EDs [3]. The new guidelines for the healthcare setting advocate for the use of written or oral HIV pre-test information to streamline the testing process. Given the many limitations of giving HIV pre-test information in oral or written form to patients, we examined if a video could be a good substitute for an in-person discussion.

We found that ED patients randomly assigned to the educational video, "Do you know about rapid HIV testing?" demonstrated as good or better comprehension of rapid HIV testing fundamentals than ED patients randomly assigned to an in-person discussion. The video we developed and evaluated is freely available via the internet [10]. We believe that it can be used as a substitute for an in-person discussion for persons undergoing rapid HIV testing, whether in the ED or other settings. This substitution is particularly important for resource poor, busy, rapid HIV testing settings with limited numbers of or access to HIV counselors.

We also observed that patient knowledge about rapid HIV testing can clearly be improved in the short term through an in-person discussion. Our findings also show that patients who do not receive any type of rapid HIV pre-test information have much less knowledge about HIV, HIV transmission, HIV prevention, and HIV testing than those who undergo an in-person discussion with an HIV test counselor. It is even more concerning that this knowledge was low even though the majority of patients had previously been tested for HIV and presumably received HIV pre-test information as part of that test. Since rapid HIV testing involves a few nuances compared to standard HIV testing, particularly the three possible test results, our results suggest that some type of information should be provided to patients prior to their undergoing testing. Our study results indicate that either an in-person discussion or a video presentation would satisfy this need. Of course, the long-term retention of this knowledge was not directly assessed through this study. Future studies could evaluate whether or not the knowledge was retained, which might impact how the information is used to prevent HIV transmission.

This study has a number of limitations. First, the study was conducted at a single ED in the US and had several exclusion criteria that reduced the scope of the study population. As a result, the results might not be generalized to other populations and settings. However, given the diversity of the participants in our study, the random selection of participants, and the random assignment of participants to information arms, we believe that our results are reasonably applicable to most other English-speaking patient groups. Second, the questionnaire we developed and employed in this study is not a perfect indicator of patient comprehension of these topics. There might have been other areas of difference or even similarity between arms not measured in our questionnaire. However, the administration of an identical questionnaire in a standardized fashion to randomly selected groups helped ensure that it was a consistent evaluator. Further, the extensive questionnaire development process helped produce a reasonably valid estimator of patient knowledge on the presented topics. Third, the study was not conducted on patients undergoing rapid HIV testing. It is possible that patients who know they will be tested would have a greater stake in listening to the information presented to them. As a result, differences between the in-person discussion and video arms might be reduced for these patients. To address this possibility, we are conducting a larger study comparing in-person discussion to video presentation among patients being tested for HIV OraQuick^®^. Fourth, the study did not show that members of the video arm had greater mean scores than those in the in-person discussion arm. A much larger sample size might have demonstrated a difference. Further, there is some loss of power to using a Wilcoxon rank-sum test, which might account for a failure to detect a difference between the video and in-person discussion arms. Also, because we originally assumed a normal distribution of scores and planned our sample size for trial 2 accordingly, we might have underpowered our study. A non-inferiority study design might have been a preferable method for evaluating this trial. For the subsequent larger trial, we changed the trial design to accommodate a non-inferiority design. Fifth, the randomization process did not produce equal size groups and was not concealed to the RA. Although no significant loss of power is expected, given the sample size, there is a possibility that the RA could have encouraged some patients to be in the trial based upon the type of assignment (video, in-person discussion, or no information) they were intended to receive. We expect that this influence is probably small and that more than likely the imbalance is due to less than optimal allocation of patients into groups.

## Conclusion

We found that the video "Do you know about rapid HIV testing?" appears to be an acceptable substitute for an in-person discussion on rapid HIV testing with OraQuick^® ^in the ED setting. We also observed that either form of pre-test information was preferable than providing no information. We believe that this freely available video can be used for patients undergoing rapid HIV testing with OraQuick^® ^to help inform them about HIV and rapid HIV testing particularly in resource poor or busy HIV testing settings, such as the ED.

## Abbreviations

HIV: human immunodeficiency virus

Tablet PC: personal computer

CDC: Centers for Disease Control and Prevention

ED: emergency department

US: United States

AIDS: acquired immunodeficiency syndrome

IRB: institutional review board

RA: research assistant

## Competing interests

The author(s) declare that they have no competing interests.

## Authors' contributions

RCM conceived of, designed, and executed the study, performed the analysis, and was the primary author of this manuscript. EMG assisted with the design, execution, and analysis of the study. MAC, KHM, GRS, and VGD were mentors to RCM on this project and helped oversee the design and analysis of the study and the preparation of the manuscript. All authors read and approved the final manuscript.

## Pre-publication history

The pre-publication history for this paper can be accessed here:



## Supplementary Material

Additional file 1 Development of the rapid HIV testing video, "Do you know about rapid HIV testing?". This manuscript provides a detailed summary of the development of the rapid HIV testing video, "Do you know about rapid HIV testing?" Click here for file

Additional file 2 Development of the "HIV pre-test information" questionnaire. This manuscript provides a detailed summary of the development of the "HIV pre-test information" questionnaire used for this trial. Click here for file
