# Supplementary material for: Comparison of patient comprehension of rapid HIV pre-test fundamentals by information delivery format in an emergency department setting
Source: BMC Public Health. 2007 Sep 12;7:238. doi: 10.1186/1471-2458-7-238 (PMC2080636; doi:10.1186/1471-2458-7-238)
Supplement: Additional file 1 — Development of the rapid HIV testing video, "Do you know about rapid HIV testing?". This manuscript provides a detailed summary of the development of the rapid HIV testing video, "Do you know about rapid HIV testing?" [file 1471-2458-7-238-S1.pdf]

Development of the Rapid HIV Testing Video, "Do you know about rapid HIV testing?"

Introduction

This paper describes the process of creating an animated and live-action educational video specifically for rapid HIV testing, entitled, "Do you know about rapid HIV testing?"[10] The freely available video includes Centers for Disease Control and Prevention (CDC) suggested elements of pre-test HIV information[11] and educates patients about the process and test results of rapid HIV testing with OraQuick®. The goal of this paper is to outline the background research, creative process, development steps, and assessment procedures involved in video development as well as the costs, time involved, successes, and lessons learned to create and evaluate the video.

Background Research

We initially hoped to find an existing educational video on HIV testing that could be adapted as an alternative to an in-person discussion of rapid HIV pre-test information. During August-October 2004, we searched for HIV testing educational videos, video scripts, and related studies through direct inquiries to international, national, state, and local health and HIV organizations; electronic medical literature searches; web-browser internet searches; research meeting abstract searches; hand searches of references and of medical journals; and consultations with professional colleagues. Table 1 shows the sources and places searched, methods of searching, and the yield of the searches.

<u>Video Evaluations</u>

We reviewed the compiled list of videos and descriptions of their content. Due to concern that older videos might not contain current information about HIV and HIV testing, we limited

our subsequent video evaluations to three recent commercially available videos[12-14], a set of four videos from a research study at Yale University[15, 16], and an Abbott Diagnostics OraQuick® marketing video designed to provide instruction on how to perform the rapid HIV test.[17] We were not able to obtain any video scripts to review.

We used the Instructional Video Evaluation Instrument created by Beaudin and Quick[18] as the general guide in evaluating the videos. We assessed the videos along three main areas: content, organization, and technical quality. Next, we established additional criteria that were specific to the ultimate goal of using the video as a substitute for an in-person discussion on rapid HIV testing. First, the video needed to meet CDC recommendations regarding the content of HIV pre-test information.[11] Second, the language of the video should be no more complex than a 7th-8th grade reading comprehension level. Third, the video must be appealing and relevant to men and women, multiple racial/ethnic groups, and viewers between the ages of 18 and 55 (the age range of adults most likely to be tested for HIV). Finally, the video should be sufficiently brief to be viewed in a single, short testing encounter.

Although there were several excellent videos, none met all of the aforementioned criteria. The reasons the videos did not meet criteria included having poor visual aids or sound quality; inappropriate length; high English-level comprehension levels; poor production quality; and age-specific or demographic-specific content. In addition, with the exception of the Abbott Diagnostics OraQuick® video[17], none had information on rapid HIV testing. Unfortunately the OraQuick® video's focus was on test administration and did not contain the CDC-recommended elements of HIV pre-test information.

Video Creation

Because there were no educational videos appropriate to adapt to our needs, we decided to create our own video. Although we had the requisite scientific knowledge to develop the educational content of the video, we did not have the technical capabilities to produce a high-quality video. Since Abbott Diagnostics had already produced an instructional video on OraQuick® and had the required technical expertise and resources, we formed a partnership with them to create a new video. The partnership agreement specified that Abbott would produce the video. This production included advising the researchers on the script and video format, creating images and graphics, providing the audio needs for the video, and compiling and editing the intermediate and final versions of the video. In exchange, we (the researchers) would compose the written script, provide the scientific and technical assistance for the video's content, conduct pilot testing of the video, edit its content, and test the video's effectiveness in a randomized, controlled clinical trial. As part of the agreement, Abbott Diagnostics would own the video and we would be able to use it for rapid HIV testing. In addition, both partners agreed to independently secure and oversee funding for their portions of the project. As a result, no funds were exchanged between Abbott Diagnostics and the researchers.

Drafting the Script

The outline for the video script addressed five broad thematic areas: the definition of HIV and AIDS; HIV transmission; HIV prevention; the underlying concepts of HIV testing and the benefits and consequences of being tested; and the features, conduct, and meaning of rapid HIV test results using the OraQuick® product. The script comprised five sections that corresponded to the thematic areas. An outline of the concepts addressed within each area for the video is in Figure 1. These five themes were designed to be presented in a progressive, iterative manner that

ultimately led to the final topic – undergoing testing with OraQuick®. We decided to present the concepts within these five themes in a didactic yet conversational form that would mimic an in-person discussion between an HIV test administrator and an individual being tested. To that end, we wrote the script as if the narrator was speaking directly to the viewer using the second person subject form (implicitly or explicitly, as needed). Also, we began with an overview of the objectives and a few introductory questions for each of the five main sections just as an HIV test administrator might do in a one-on-one session. We drafted the script to be understood by viewers with a 7th-8th grade reading comprehension level, as measured by the Fleisher-Kincaid scale incorporated in Microsoft Word® (Microsoft Corporation, Redmond, WA).

Selection or Creation of Visual Images

After reviewing our text, the Abbott Creative Network partners suggested that the video would be more effective as a learning tool if animated characters portrayed as undergoing a rapid HIV pre-test information session were included. We decided to use a human young-adult male-female pair of animated characters as our "protagonists." We believed that having one pair of primary characters continued throughout the video would promote continuity of the storyline. We also reasoned that in the widespread usage of the video, most of the viewers would young, adult, heterosexuals, and thus might more easily identify with primary characters like them. To address concerns that some viewers might not feel that the characters represented them, we included ancillary animated characters in the video who were single or in male-male or female-female pairings.

Because we wanted our viewers to consider the animated protagonists as representing them, we created racially and ethnically ambiguous characters. We appreciated that these

characters did not represent all racial and ethnic groups, but hoped they appeared to be a member of one or more of the major racial or ethnic groups in North America. The animators created six different male and female characters with different racial or ethnic features, hair styles, and skin colors. We presented these characters to a convenience sample of twenty employees at our institution who identified themselves as black or Hispanic or of a mixed-race/ethnic background. With their assistance, we chose a male and female pair who could possibly be of black, Hispanic, white, Middle Eastern, Mediterranean, Native American, or Indian ethnicity or race. We did not assign names to our characters for several reasons: to simulate testing confidentiality, to avoid distraction from the purpose of the video, to help viewers picture themselves as one of the characters, and to allow for the racial/ethnic ambiguity of our characters. To achieve an even wider ethnic or racial mix for the animated characters, we included ancillary characters who could be from racial or ethnic backgrounds different than the animated protagonists.

To help illustrate key terms from the script, the animators selected words or short phrases and presented them on the video screen simultaneously with the narration. The importance of these words or phrases was emphasized with bold text, flashing or moving images, unique shapes, or other features. The animators also created several graphics, images, and short features that stressed key concepts. These included silhouetted images of HIV being transmitted from one figure to another, a human figure forming antibodies in response to an HIV infection, and a clock that counted the twenty minutes it would take for an OraQuick® test to be performed. In addition to the animation, the video had live-action images of people in everyday life and of patients undergoing rapid HIV testing with OraQuick®.

Production of Draft Video

The video production team created a draft storyboard that showed the proposed images alongside the script. We revised the storyboard with the assistance of HIV testing experts. The video production team created several proposed images and graphics which were also reviewed and edited as needed. There were several cycles involved in the process of reviewing, editing, and revising the text and storyboard until a draft was ready for final production. Our video production partners chose a male professional narrator with an older, authoritative, yet non-threatening voice, much like those used for documentary films. The final draft of the video was 9.5 minutes in length.

Audience Assessment and Initial Pilot Testing of the Draft Video

We conducted individual intensive patient interviews to gain perspectives on the video from the intended audience---patients who might undergo rapid HIV testing in the emergency department. The goals and questions for these interviews are shown in Figure 2. During April 2005, one of the co-authors enrolled patients for the individual intensive patient interviews in the urgent care and ambulatory care areas of our emergency department who were age 18-55; not pregnant; not a prisoner or in home confinement; not known to be HIV infected; not participating in an HIV vaccine study; able to speak, read, and write in English; not deaf; and not critically ill, intoxicated, or presenting to the ED for a psychiatric problem. For this qualitative appraisal, we sought volunteers from diverse demographic groups. We planned to enroll a total twenty emergency department patient volunteers according to these demographic criteria: approximately ten men and ten women; an equal number of people from five age groups (18-25, 26-35, 36-45, 46-55); an equal number of white and non-white patients; and approximately one-third each who

finished, did not finish, or studied beyond high school. Participants received a $25 gift card to a local pharmacy in an expression of gratitude for their assistance.

The individual patient interviews involved asking participants to watch the video on a Tablet personal computer while they were in the emergency department. Next, the patient was asked to answer a set of questions about their impressions of the video (Figure 2). All interviews were conducted by the same interviewer. Participant responses were recorded via audio tape. In addition, the interviewer recorded all her observations, written notes of the participant responses, and any additional non-verbal responses by the participant. The audio tapes were transcribed, combined with the written notes, and reviewed by the study authors.

We approached thirty-one emergency department patients who appeared to meet study criteria. Eleven declined to participate. The final sample included four people in each of the aforementioned five age groups, ten men and ten women, ten whites and ten non-whites, and five who did not finish high school, six who finished high school, and seven who studied beyond high school. With the assistance of CDC and other HIV testing experts, we analyzed the transcripts and notes from the intensive individual patient interviews. The participants provided feedback on areas in which the video could be improved, sections that needed clarification, portions that might be changed or omitted, and narration or images that required adjustments. We incorporated the feedback and our observations from the interviews into a revised video.

Production of Final Video

Using data from the aforementioned video evaluations and the results of the pilot study (described in the accompanying paper), we implemented a set of improvements to the video. These improvements included removal of potentially offensive or confusing terms, the addition

of even more demographically diverse animated characters and images of real people, the clarification of person-person transmission and immune system response to HIV, the enhancement of several graphics to include better and simpler explanations of the topics presented, and the addition of oral sampling for rapid HIV testing. The final video is freely available for public use at (http://www.brown.edu/Departments/BRUNAP/aids_video.html).

Video Production Financial Expenditures

The estimated direct costs for creating, producing, and testing the video are provided in Table 2. Not included in the estimates are the indirect and incalculable costs that include miscellaneous supplies, overhead, general and statistical software costs, desktop personal computer usage, access to online journals and the internet, time and effort in applying for grants to support the project, administrative paperwork and institutional review board applications, and consultation with experts. The costs and work-hours in Table 2 also do not include the costs and work hours of the development of the questionnaires utilized in the pilot study.

Table 1 Methods and Yield of Educational Videos and Related Studies on HIV Testing

**Source**	*International, Governmental, and Non-governmental Health Organizations*	*Medical Literature (Electronic) MEDLINE*	*Web-browser Internet Searches (Google®, Inc.)*	*Other Searches*
**Search Locations**	*Organizations included:*<br>▪ World Health Organization<br>▪ CDC<br>▪ National Institutes of Health<br>▪ National Alliance of State and Territorial AIDS Directors<br>▪ Ten Health Departments*<br>▪ American Red Cross<br>▪ Gay Men's Health Crisis<br>▪ San Francisco AIDS Foundation	*Search terms:*<br>▪ HIV (educational, test, testing) video<br>▪ AIDS (educational, test, testing) video<br>▪ HIV intervention<br>▪ AIDS intervention	*Search terms:*<br>▪ HIV (educational, test, testing) video<br>▪ AIDS (educational, test, testing) video<br>▪ HIV video catalogue<br>▪ AIDS video catalogue<br>▪ HIV intervention<br>▪ AIDS intervention	▪ Searches of journal websites for unpublished abstracts or published articles not located in other searches<br>▪ Annals of Emergency Medicine<br>▪ Academic Emergency Medicine<br>▪ American Journal of Emergency Medicine<br>▪ Emergency Medicine Journal<br>▪ Journal of Emergency Medicine<br>▪ AIDS<br>▪ AIDS Education and Prevention<br>▪ Journal of AIDS<br>▪ HIV and Behavior<br><br>▪ Searches of conference websites<br>▪ 2003 CDC National HIV Prevention Conference<br>▪ 2004 International AIDS Conference<br>▪ 2004 Infectious Disease Society of America Annual Meeting<br><br>▪ Colleague inquiries
**Search Methods**	Website searches, telephone calls, letters, emails to the organizations	▪ MEDLINE searched for English-language abstracts, 1966-2004<br>▪ Abstracts reviewed<br>▪ Relevant articles reviewed<br>▪ Study authors contacted via email, telephone, and letters	▪ Internet searched for English-language videos primarily for adults<br>▪ Video catalogues reviewed<br>▪ Video descriptions reviewed for current HIV testing videos<br>▪ Distributors/producers of videos contacted	▪ Websites searched for abstracts of studies or reports on HIV testing videos<br>▪ Colleagues involved in HIV testing and emergency medicine inquired about HIV testing involving videos
**Search Yield**	Two videos on HIV testing	60 abstracts → 15 relevant articles<br>→ 11 authors located and contacted<br>→ 4 related videos on HIV testing	▪ 17 video producers/distributors<br>→ 7 retrievable catalogues<br>▪ 29 HIV video names located<br>→ 8 videos on HIV testing<br>→ 5 retrievable videos<br>→ 3 recent/current videos<br>▪ One training video on OraQuick® for health care providers<br>▪ One research abstract on video-faciliated HIV testing<br>▪ One thesis on video development and assessment of HIV treatment	▪ No additional abstracts located<br>▪ No additional videos found

*Ten state/territory health departments of states/territories with the highest AIDS prevalence: New York, California, Florida, Texas, New Jersey, Pennsylvania, Illinois, Georgia, Maryland, and the District of Columbia

Figure 1 Outline of Video Concepts

HIV/AIDS

- Definitions of HIV and AIDS
- Relationship of HIV to AIDS
- HIV's destruction of the immune system and its consequences
- Time of progression from HIV to AIDS

HIV Transmission

- Person-to-person transmission of HIV
- Common modes of HIV transmission
- Ways HIV is not transmitted
- Universal ability of people to be infected with HIV
- Increased risk of being infected with HIV with repeated exposures

HIV Prevention

- Ways to prevent HIV infection
    - Condom usage and abstinence from sex
    - Avoidance of injection-drug usage, avoidance of injection-drug equipment sharing
    - Avoidance of breast feeding by HIV-infected women

HIV Testing and Benefits and Consequences of HIV Testing

- Benefits of being tested for HIV: reduced anxiety, discover if infected, earlier access to treatment, prevention of transmission to others, encourage contacts to get tested
- Consequences of being tested for HIV: stressful waiting for results, feelings of isolation, and financial and social concerns
- Lack of vaccine or cure for HIV, but availability of helpful treatments
- HIV antibodies as a marker of HIV infection
- "Window period" of 3-6 months for detection of antibodies through HIV tests and its implications on testing for HIV
- Description of two types of HIV tests, standard and rapid
- Advantages of rapid HIV tests

Rapid HIV Testing with OraQuick®

- Procedures for conducting the rapid HIV test
- Three possible rapid HIV test results (negative, preliminary positive, and invalid) and their meanings
- Need for confirmatory testing for preliminary positive test results
- Need for additional testing for invalid test results

Figure 2 Goals and Questions for the Audience Review of the Video Draft

Question 1: [OVERALL IMPRESSIONS]

Goal of Question 1: In addition to being an introduction to the intensive interview process, to determine the participant's primary or overall response or reactions to the video

- *What were your first impressions when you saw the video?*
- *What did you like best?*
- *What did you like least?*

Question 2: [AGE APPROPRIATENESS OF PRESENTATION]

Goal of Question 2: To determine if participants believe the style and content of the video presentation are appropriate for the intended adult audience

- *How successful is the video in reaching adults between the ages of 18-55?*
- *Is it age appropriate?*
- *In what ways was it appealing or not appealing to someone in your age group?*

Question 3: [GENDER AND ETHNICITY APPROPRIATENESS]

Goals of Question 3: To determine if participants believe if the style and content of the video are applicable and appealing to both men and women and minority audiences

- *In what ways is the video appealing/not appealing to men/women like you?*
- *Or to your ethnic/racial/minority group?*

Question 4: [QUALITY AND APPROPRIATENESS OF IMAGES]

Goals of Question 4: To determine if participants believe the video images were helpful in communicating the points of the message and to determine if participants considered the images to be repellent or off-putting from the message of the video.

- *Tell me which images helped make the points in the video either clearer or more confusing.*
- *Which images, if any, were distracting?*
- *Which images, if any, were alarming or frightening?*
- *Was there anything about the characters that you found not to your liking? Anything about how they way the characters were presented that you didn't like?*

Question 5: [QUALITY OF AUDIO NARRATION]

Goals of Question 5: To determine if the participants believed the audio narration was comprehensible and engaging, in terms of clarity, speed and style

- *What did you think about the announcer's voice, in terms of clarity, speed, and style?*
- *How easy or difficult was it to listen to his voice?*

Question 6: [LENGTH OF VIDEO]

Goal of Question 6: To determine if the participants believed the length of the video was appropriate in regards to delivery of the message

- *What did you think about the length of the video?*
- *Was it too long, too short, or just right?*
- *At what point, if at all, did you notice yourself becoming distracted or bored?*
- *How about more interested?*

Question 7: [QUALITY AND COMPREHENDABILITY OF TOPICS ADDRESSED]

Goals of Question 7: To determine if participants believed the messages of the video were expressed clearly and the messages were pertinent and adequate to the underlying purpose of the video (rapid HIV testing pre-test information)

- *What parts of the video did you understood well?*
- *What parts of the video did you not understand?*
- *What information was especially helpful?*
- *What was not helpful?*
- *Is there anything else you would like to know about rapid HIV testing that wasn't included in the video?*
- *What about the organization and format of the video helped you understand the concepts of HIV and rapid HIV testing? What about the organization and format made the concepts more confusing?*

Question 8: [THOROUGHNESS OF TOPICS ADDRESSED]

Goal of Question 8: To determine if participants believed the explanations of the topics covered of the video were thorough or insufficient and if they were expressed redundantly

- *Can you talk about any points which were repeated unnecessarily?*
- *Can you talk about any topics you wished the video had gone over again?*

Question 9: [PRIORITIZING CRITICISMS FOR VIDEO IMPROVEMENT]

Goal of Question 9: In addition to allowing the participant to summarize responses, to determine the participants suggestions for improvement of the video

- *If you could change one thing to improve the video, what would it be and why?*

Table 2 Video Creation and Production Financial Expenditures

**Video research, creation, and production labor**	Expenses ($US)	Work-hours (estimated)
Principal investigator	22,875	480
Research assistant	8174	500
**Video cognitive assessment labor**		
Principal investigator	1430	30
Research assistant	980	60
**Video pilot testing labor**		
Principal investigator	22875	480
Research assistant	7847	480
**Abbott video production labor**	43,000	225
**Equipment and supplies**		
Two tablet PCs	4800	
Tape recorder	120	
Multi-directional microphone	25	
Gift cards to participants	250	
**Total**	112,376	2255

References

1. Branson BM, Handsfield HH, Lampe MA, et al. Revised recommendations for HIV testing of adults, adolescents, and pregnant women in health-care settings. *MMWR Recomm Rep.* Sep 22 2006;55(RR-14):1-17.
2. Wells JA. Readability of HIV/AIDS educational materials: the role of the medium of communication, target audience, and producer characteristics. *Patient Educ Couns.* Dec 1994;24(3):249-259.
3. Wells JA, Ruscavage D, Parker B, McArthur L. Literacy of women attending family planning clinics in Virginia and reading levels of brochures on HIV prevention. *Fam Plann Perspect.* May-Jun 1994;26(3):113-115, 131.
4. Mitchell K, Nakamanya S, Kamali A, Whitworth JA. Community-based HIV/AIDS education in rural Uganda: which channel is most effective? *Health Educ Res.* Aug 2001;16(4):411-423.
5. Sherr L, Hedge B. The impact and use of written leaflets as a counselling alternative in mass antenatal HIV screening. *AIDS Care.* 1990;2(3):235-245.
6. Akkad A, Jackson C, Kenyon S, Dixon-Woods M, Taub N, Habiba M. Informed consent for elective and emergency surgery: questionnaire study. *Bjog.* Oct 2004;111(10):1133-1138.
7. Ley P. *Communicating with patients. Improving communication, satisfaction and compliance.* London: Croom Helm; 1988.
8. Arthur VA. Written patient information: a review of the literature. *J Adv Nurs.* Jun 1995;21(6):1081-1086.
9. Calderon Y, Haughey M, Bijur PE, et al. An educational HIV pretest counseling video program for off-hours testing in the emergency department. *Ann Emerg Med.* Jul 2006;48(1):21-27.
10. Merchant R, Gee E, Christie SI. Do you know about rapid HIV testing? Available at: http://www.brown.edu/brunap; 2005.
11. Centers for Disease Control and Prevention. Revised guidelines for HIV counseling, testing, and referral. *MMWR Recomm Rep.* 2001;50(RR-19):1-57.
12. AIMS Multimedia. HIV/AIDS 101; 2003.
13. Pyramid Film and Video. HIV and AIDS; 1992.
14. Human Relations Media. The HIV Test: Who Should Take It? What Does It Show?; 1993.
15. Kalichman SC, Cherry C, Browne-Sperling F. Effectiveness of a video-based motivational skills-building HIV risk-reduction intervention for inner-city African American men. *J Consult Clin Psychol.* Dec 1999;67(6):959-966.
16. Kalichman SC, Coley B. Context framing to enhance HIV-antibody-testing messages targeted to African American women. *Health Psychol.* May 1995;14(3):247-254.
17. *When waiting is not an option...* Chicago: Abbott Diagnostics; 2003.
18. Beaudin BP, Quick D. Instructional video evaluation instrument. *Journal of Extension.* 1996;34(3).
