# Supplementary material for: Comparison of patient comprehension of rapid HIV pre-test fundamentals by information delivery format in an emergency department setting
Source: BMC Public Health. 2007 Sep 12;7:238. doi: 10.1186/1471-2458-7-238 (PMC2080636; doi:10.1186/1471-2458-7-238)
Supplement: Additional file 2 — Development of the "HIV pre-test information" questionnaire. This manuscript provides a detailed summary of the development of the "HIV pre-test information" questionnaire used for this trial. [file 1471-2458-7-238-S2.pdf]

Development of the "HIV Pre-Test Information Comprehension" Questionnaire

Introduction

This paper describes in detail the process of creating and assessing the "HIV pre-test information comprehension" questionnaire used in the pilot trial described in the accompanying paper. The goal of this paper is to outline the background research, development steps, and assessment procedures involved in the questionnaire development as well as successes and lessons learned to create and evaluate the questionnaire.

Background Research

We first searched for and reviewed prior studies on and previously used questionnaires about HIV and HIV testing knowledge in many different settings. We hoped to use questions from these prior questionnaires in their present form or as modified for the needs of this study. Table 1 summarizes the types of searches conducted and the yields of those searches. The review of the questionnaires and studies included an examination of the quality of the questions, the rigor of the survey development, the format of the questions and responses, the survey administration techniques, the populations studied, the purposes for conducting the study, and the study results.

Questionnaire Development

The development of the questionnaire proceeded in three main phases: (1) question selection, composition, and refinement; (2) initial pilot testing; and (3) cognitive-based assessments of questions through intensive patient interviews.

Question selection, composition, and refinement

Using the results of our review of prior studies and questionnaires located in the background research, we first outlined the goals for the questionnaire, considered which possible topics could be included, and decided upon a format for the questionnaire (Table 2). Because the questions would be delivered orally to the respondent, we determined that the best format of the questions and responses was for the question to be in the form of a statement and the responses to be “true”, “false”, and “don’t know”. We reasoned that short statements would be easier for the respondent to comprehend and that uniform responses would help the respondent focus on the statements and not try to choose from a varying list of response options. Further, short statements would be easier and quicker to deliver orally.

Next, we generated lists of potential questions for the questionnaire using questions located during the background research. There were no available questions that directly compared video or other alternative methods of HIV pre-test information delivery. Few surveys had questions on HIV testing. As a result, we primarily considered questions from surveys that assessed HIV and AIDS knowledge.[9-15]

For questions that could potentially be adapted, we revised their form and content. These adjustments included changing the form of the question or response; updating the content of the questions to reflect current understanding of HIV or other topics; reducing the questions to single rather than multiple parts; paring down the question stem or response for brevity; reformatting the question or responses; and adjusting the language, vocabulary or syntax to improve participant comprehension of the question. If there were no suitable questions for a given topic, we composed new questions.

Once we formed the list of potential questions, we critically reviewed and revised them to ensure that the content was correct and complete; the grammar, syntax, and comprehension level were appropriate; they could be delivered orally and comprehended readily in that form; they were applicable to the study goals; and they were devoid of problems common to many survey questions (were not value-laden, did not contain jargon or slang, were not double-barreled in content, did not have double-negatives, etc.). We administered the questions to colleagues and staff members and further revised them based upon their comments and our observations.

Initial pilot testing

After forming draft questions for the "HIV pre-test information comprehension" questionnaire, we conducted an initial pilot test of the instrument. The overall goals of the initial pilot testing were to: (1) identify problems in the wording of the questions or responses, (2) gauge the reactions participants had to our questions, (3) determine the length of time it took to deliver the survey, (4) estimate the number of patients who might complete the questionnaires, and (5) uncover any topics that we were failing to address.

The primary goal was to find questions that could assess participant understanding of the pre-test information they received (video vs. in-person discussion). To that end, in the initial pilot testing, we intended to eliminate questions that were not good discriminators of participant knowledge. Modifying the suggestions of Carey, et al., we decided to eliminate questions that were answered correctly by fewer than 20% or greater than 80% of the respondents.[11, 12] We planned to have at least 25 questions in the final version to balance the need for statistical stability in our analyses (more questions) with participant tolerance (fewer questions) and to have approximately four to five questions within each of the five categories outlined in Table 2.

Methods and conduct of initial pilot testing

The pilot testing proceeded in three iterations. Each iteration consisted of presenting the draft questionnaires to a group of ten participants. After each iteration, the questions were modified as needed. Some questions were revised, others eliminated, and new ones were written to create a new version of the questionnaires before the start of the next iteration. The worst performing questions were eliminated (those answered by greater than 80% or fewer than 20% of the participants). The stem of the true and false questions for this questionnaire were adjusted after each iteration so that there would be a balance between the number of questions for which true and false were the correct responses . For example, the stem for a question for which the correct response was "true" would be changed so that the correct response would be "false." The sequence of questions for this questionnaire was also randomly re-ordered after each iteration with the assistance of a random number sequence generator available at www.random.org (Trinity College, Dublin, Ireland).

Each of the three iterations included ten patients, five women and five men, from a convenience sample of ED patients awaiting medical care in the urgent care and the ambulatory care sections of the ED. Patients 18-55-years-old were eligible to participate in the pilot testing if they were not pregnant; not an inmate; not known to be HIV infected; could speak, read, and write in English; were not deaf; or were not critically ill, intoxicated, or presenting to the ED for a psychiatric problem. This and all parts of the study were approved by the hospital institutional review board. Participants received a $2 gift card to a local pharmacy.

A research assistant (RA) screened medical records of patients awaiting medical care in the ED during 9 a.m. to 5 p.m. weekdays during February 2005. Participants eligible for the study were first administered a semi-scripted in-person discussion with the RA on five topics:

HIV and AIDS, HIV transmission, prevention, testing, and rapid HIV testing. An outline of the points covered in the in-person discussion is provided in the accompanying article. The outline was created by the study authors and is based upon the 2001 CDC recommendations for pre-test information[19] with additional information on rapid HIV testing specific to OraQuick®[20]. The RA was certified by the state to conduct HIV test counseling and underwent approximately twenty hours of training and mock-interviews to practice delivering the in-person discussion prior to conducting the pilot study.

Results of initial pilot testing

The RA approached 42 patients of whom twelve declined to participate. Fifteen of the participants were women and fifteen were men. We presented 28 questions in the first iteration, 32 in the second, and 32 in the third iteration. During the first iteration, we reformatted one question that was poorly worded, eliminated twelve questions from consideration because 90% or more of respondents answered them correctly, and removed another question because it could be answered correctly in different ways depending upon participant interpretation of the question. In the second iteration, we reformatted six poorly worded questions, changed the focus of another question, eliminated four questions because 90% or more of respondents answered them correctly, and removed another question because it could be answered correctly in different ways, depending upon how it was interpreted. In the last iteration, we did not eliminate any questions.

Cognitive-based assessments of "HIV pre-test information comprehension" questionnaire

The initial pilot testing of the questionnaire did not determine whether or not the participants truly understood the questions or whether they answered the question in the way it

was intended to be answered. We conducted cognitive-based assessments of the questionnaires to address these concerns based upon interviewing techniques suggested by other researchers.[21-23] The overall goals for the cognitive-based assessments were to understand participant thought processes in answering of the questions, obtain their impressions of the questions, discover participant difficulties in understanding the questions or particular words in the questions, identify concerns about the question content and phrasing, and uncover any demographic or cultural-related inappropriateness in the questionnaire

Methods and conduct of cognitive-based assessments

First, we reviewed our questionnaires and identified what we believed to be concepts, questions, phrases, expressions, and words that might be difficult for participants to understand. Second, we established specific goals for our cognitive-based assessments based upon these identified areas of concern. Third, we composed probe questions that would address these concerns. Examples of the survey questions, the goals, and the corresponding cognitive probes, are provided in Table 3.

For this qualitative analysis, we strove to include a mixture of patients from different demographic groups to obtain a broader range of participant input. We selected patients using a quota system based on demographic characteristics (age, gender, and years of education) that would provide a diversity of participants. The participant exclusion criteria established in the initial pilot testing was also used for the cognitive-based assessments. At the completion of the intensive interviews, participants were provided an envelope with a thank you letter, a $10 gift card to a local pharmacy, and an educational brochure on HIV and HIV testing that contained a list of HIV testing centers in the area.

Two RAs worked in tandem to conduct the cognitive assessments. One RA read the survey questions from the Tablet PC QDS program and entered the participant responses. The second RA read the cognitive interview questions and recorded the respondents' answers and any observations on paper. In addition to collecting participant verbal responses to the questions, the second RA recorded non-verbal responses to the questions, participant requests for clarifications, pauses in responses, facial responses, and the prefacing of answers.

Results of cognitive-based assessments

The RAs interviewed patients in March 2005. Each interview lasted approximately 35-45 minutes. The final sample included six women and four men; two patients in each of five age groups; three patients who had not completed twelve years of formal education, four who had twelve years of formal education, and three who had more than twelve years of formal education. Two more patients initially agreed to participate, but declined to finish the survey.

Cognitive-based assessments of the questionnaire were enlightening. There were errors in questions that had performed well in the initial pilot study and were presumed to be good discriminators of participant knowledge of HIV pre-test information fundamentals. For example, a question about a link between tattoos and HIV transmission could have been correctly answered as false or true depending upon participant assumptions about the context and purpose of the question. This question was subsequently eliminated from the survey. A question that asked about the ability of bleach to destroy HIV in the context of injection-drug usage was modified based upon participant feedback. Participants had trouble with the statement that bleach "killed" HIV and were more comfortable with the alternative statement of bleach "destroying" HIV. Using the results of cognitive-based assessments, we made several modifications to the questionnaire. The resultant questionnaire is provided in the accompanying article.

References

1. Branson BM, Handsfield HH, Lampe MA, et al. Revised recommendations for HIV testing of adults, adolescents, and pregnant women in health-care settings. *MMWR Recomm Rep.* Sep 22 2006;55(RR-14):1-17.
2. Rhodes KV, Gordon JA, Lowe RA. Preventive care in the emergency department, Part I: Clinical preventive services--are they relevant to emergency medicine? Society for Academic Emergency Medicine Public Health and Education Task Force Preventive Services Work Group. *Acad Emerg Med.* Sep 2000;7(9):1036-1041.
3. Rothman RE, Ketlogetswe KS, Dolan T, Wyer PC, Kelen GD. Preventive care in the emergency department: should emergency departments conduct routine HIV screening? a systematic review. *Acad Emerg Med.* Mar 2003;10(3):278-285.
4. Babcock Irvin C, Wyer PC, Gerson LW. Preventive care in the emergency department, Part II: Clinical preventive services--an emergency medicine evidence-based review. Society for Academic Emergency Medicine Public Health and Education Task Force Preventive Services Work Group. *Acad Emerg Med.* Sep 2000;7(9):1042-1054.
5. Merchant R, Gee E, Christie SI. Do you know about rapid HIV testing? Available at: http://www.brown.edu/brunap; 2005.
6. Centers for Disease Control and Prevention. National Health and Nutrition Examination Survey Questionnaire. Atlanta, GA: U.S. Department of Health and Human Services, Centers for Disease Control and Prevention; 2002.
7. Centers for Disease Control and Prevention. Behavioral Risk Factor Surveillance System Survey Questionnaire. Atlanta, GA: U.S. Department of Health and Human Services, Centers for Disease Control and Prevention; 2004.
8. Centers for Disease Control and Prevention. National Health Interview Study Survey Questionnaire. Atlanta, GA: U.S. Department of Health and Human Services, Centers for Disease Control and Prevention; 2004.
9. Gyarmathy VA, McNutt LA, Molnar A, et al. Evaluation of a comprehensive AIDS education curriculum in Hungary--the role of good educators. *J Adolesc.* Oct 2002;25(5):495-508.
10. Nwokocha AR, Nwakoby BA. Knowledge, attitude, and behavior of secondary (high) school students concerning HIV/AIDS in Enugu, Nigeria, in the year 2000. *J Pediatr Adolesc Gynecol.* Apr 2002;15(2):93-96.
11. Carey MP, Schroder KE. Development and psychometric evaluation of the brief HIV Knowledge Questionnaire. *AIDS Educ Prev.* Apr 2002;14(2):172-182.
12. Carey MP, Morrison-Beedy D, Johnson B. The HIV-Knowledge Questionnaire: Development and Evaluation of a Reliable, Valid, and Practical Self-Administered Questionnaire. *AIDS and Behavior.* 1997;1(1):61-74.
13. Walter EB, Royce RA, Fernandez MI, DeHovitz J, Ickovics JR, Lampe MA. New mothers' knowledge and attitudes about perinatal human immunodeficiency virus infection. *Obstet Gynecol.* Jan 2001;97(1):70-76.
14. Hancock T, Mikhail BI, Santos A, Nguyen A, Nguyen H, Bright D. A comparison of HIV/AIDS knowledge among high school freshmen and senior students. *J Community Health Nurs.* 1999;16(3):151-163.

15. Ford K, Wirawan DN, Reed BD, Muliawan P, Sutarga M. AIDS and STD knowledge, condom use and HIV/STD infection among female sex workers in Bali, Indonesia. *AIDS Care.* Oct 2000;12(5):523-534.
16. Pronyk PM, Kim JC, Makhubele MB, Hargreaves JR, Mohlala R, Hausler HP. Introduction of voluntary counselling and rapid testing for HIV in rural South Africa: from theory to practice. *AIDS Care.* Dec 2002;14(6):859-865.
17. Sy FS, Rhodes SD, Choi ST, et al. The acceptability of oral fluid testing for HIV antibodies. A pilot study in gay bars in a predominantly rural state. *Sex Transm Dis.* Apr 1998;25(4):211-215.
18. Kassler WJ, Dillon BA, Haley C, Jones WK, Goldman A. On-site, rapid HIV testing with same-day results and counseling. *Aids.* Jul 1997;11(8):1045-1051.
19. Centers for Disease Control and Prevention. Revised guidelines for HIV counseling, testing, and referral. *MMWR Recomm Rep.* 2001;50(RR-19):1-57.
20. OraSure Technologies. OraQuick Advance Rapid HIV1/2 HIV Antibody Test Package Insert. Bethlehem, PA; 2005.
21. Jobe JB, Mingay DJ. Cognitive research improves questionnaires. *Am J Public Health.* Aug 1989;79(8):1053-1055.
22. Sudman S, Bradburn NM, Schwarz N. *Thinking about answers : the application of cognitive processes to survey methodology*. San Francisco: Jossey-Bass Publishers; 1995.
23. Willis GB, Royston, Patricia, Bercini, Deborah. The use of verbal report methods in the development and testing of survey questionnaires. *Applied Cognitive Psychology.* 1991;5(3):251-267.

Table 1 Methods and Yield of Background Research

**Source**	*CDC Health-related Surveys*	*Medical Literature (Electronic) MEDLINE*	*Web-browser Internet Searches (Google®, Inc.)*	*Other Searches*
**Search Locations**	*National Center for Health Statistics website*<br><br>*Primary surveys included:*<br>▪ 2004 Behavioral Risk Factor Surveillance System (BRFSS)<br>▪ 2004 National Health Interview Survey (NHIS)<br>▪ 2002 National Health and Nutrition Examination Survey (NHANES)	*Search terms:*<br>▪ HIV and HIV test comprehension<br>▫ AIDS (diagnosis, seropositivity, serodiagnosis)<br>▫ HIV (infections, diagnosis, seropositivity, antibodies, analysis, serodiagnosis)<br>▫ HIV or AIDS	*Search terms:*<br>▪ HIV test (survey knowledge, knowledge survey)<br>▪ HIV (knowledge, knowledge survey)	▪ Searches of journal websites for unpublished abstracts or published articles not located in other searches<br>▫ *Annals of Emergency Medicine*<br>▫ *Academic Emergency Medicine*<br>▫ *American Journal of Emergency Medicine*<br>▫ *Emergency Medici*
**Search Yield**	No questions on HIV or HIV testing comprehension	▪ HIV and HIV test comprehension<br>500 abstracts → 40 relevant articles → 25 surveys available for review	▪ HIV and HIV test comprehension<br>▫ 10 additional surveys	▪ No additional abstracts located<br>▪ One unpublished article

Table 2 Questionnaire Goal, Outline, Format	
	**HIV pre-test information comprehension questionnaire**
**Goal**	▪ Ascertain the extent participants understand rapid HIV pre-test fundamentals presented in an in-person discussion or video presentation
**Outline**	▪ Definition of HIV/AIDS<br>▫ Differences between HIV and AIDS<br>▫ Nature of HIV<br><br>▪ HIV transmission<br>▫ Ways of transmission<br><br>▪ HIV prevention<br>▫ Methods of prevention<br><br>▪ HIV testing<br>▫ Nature of HIV testing<br>▫ "Window period"
**Format**	▪ Single sentence statements<br>▪ True/False/Don't Know responses
**Justifications and Considerations**	▪ Decided against multiple choice questions because of oral delivery of questions would make it difficult for respondents to remember the possible responses<br><br>▪ Decided against agree/disagree format because of possibility of acquiescence bias

Table 3 Selected survey questions and accompanying cognitive questions and goals

**Survey question**	If someone gets HIV through needle sharing, that person can only spread HIV by sharing needles.<br><br>▪ True<br>▪ False<br>▪ Don't Know	If you were HIV infected, current drug treatments would let you live longer.<br><br>▪ True<br>▪ False<br>▪ Don't Know	HIV is killed by bleach.<br><br>▪ True<br>▪ False<br>▪ Don't Know
**Cognitive assessment goals**	▪ Does the respondent understand the term "needle sharing"?<br><br>▪ Does the respondent distinguish between HIV transmission and prevention?	▪ Does the respondent understand the term "current drug treatments"?<br><br>▪ Does the respondent understand the phrase "let you live longer?"	▪ Can the respondent link needle sterilization with using bleach?<br><br>▪ Is the respondent confused by the word "killed" in this context?
**Cognitive assessment questions**	▪ What does “needle sharing” mean to you?<br><br>▪ Do you believe that someone who shares needles can infect someone with HIV other than with needles? How?	▪ What does the phrase "current drug treatments" mean to you?<br><br>▪ What does the phrase "let you live longer" mean to you?	▪ Do you believe that HIV can be killed by bleach?<br><br>▪ When might someone use bleach to kill HIV?<br><br>▪ Would your answer to this question change if we asked if HIV is "destroyed" by bleach? Or if "HIV in needles can be killed by bleach"?
